# Identification of biomarkers of Shenhailong formula in benign prostatic hyperplasia treatment: An observation study using network pharmacology and Mendelian randomization analysis

**DOI:** 10.1097/MD.0000000000045619

**Published:** 2025-11-21

**Authors:** Jia Pan, Mengyao Yu, Lihan Zhang, Jing Yin, Jie Liu, Shuwu Zhang, Peihai Zhang, Qingying Fan, Jiatong Liu, Xiaoyuan Li

**Affiliations:** a Institute of Pharmacology & Toxicology of Chinese Materia Medica, Sichuan Academy of Chinese Medicine Sciences, Chengdu, China; b Sichuan-Chongqing Joint Key Laboratory of Innovation of New Drugs of Traditional Chinese Medicine, Chengdu, China; c School of Clinical Medicine, Chengdu University of Traditional Chinese Medicine, Chengdu, China; d Department of Urology and Andrology, Sichuan Integrative Medicine Hospital, Chengdu, China; e Department of Urology and Andrology, Hospital of Chengdu University of Traditional Chinese Medicine, Chengdu, China; f Department of Pharmacy, Shandong Qingdao Huangdao District People’s Hospital, Qingdao, China; g College of Traditional Chinese Medicine, Shandong University of Traditional Chinese Medicine, Jinan, China.

**Keywords:** benign prostatic hyperplasia, biomarkers, Mendelian randomization, network pharmacology, Shenhailong formula

## Abstract

Shenhailong formula, a classic herbal prescription, shows potential in treating benign prostatic hyperplasia (BPH), but its candidate components and mechanisms remain unclear. This study aimed to preliminarily clarify its therapeutic mechanisms in BPH using network pharmacology, Mendelian randomization (MR), and molecular docking. In this study, BPH-related datasets were first obtained from Gene Expression Omnibus database, and different expression analysis was performed to identify differentially expression genes. Meanwhile, Chinese herbs related target genes and BPH-related target genes were retrieved from public database, and candidate genes were screened by taking the intersection of the 3 above. Subsequently, MR analysis was conducted to explore the causal relationship between candidate genes and BPH, and then biomarkers were generated through expression level validation. Finally, enrichment analysis, molecular regulatory network construction, and molecular docking were performed. By integrating 779 BPH-related target genes, 2879 Chinese herb-related target genes, and 3106 differentially expression genes, 41 candidate genes were obtained. MR analysis revealed that 12 genes had potential causal relationships with BPH. Eventually, 3 biomarkers were identified: serum/glucocorticoid-regulated kinase 1, epidermal growth factor, and ectonucleotide pyrophosphatase/phosphodiesterase family member 1. All 3 biomarkers were significantly enriched in the “kras signaling dn,” “pancreas beta cells,” “estrogen response early,” “estrogen response late,” and “spermatogenesis” pathways (gene set enrichment analysis, false discovery rate < 0.05). ” Furthermore, transcription factors GATA2 and JUN exerted their influence on 3 biomarkers, and the actions of hsa-miR-4459 and hsa-miR-4433a-3p were involved in the regulation of epidermal growth factor and ectonucleotide pyrophosphatase/phosphodiesterase family member 1. Additionally, molecular docking showed that Stepholidine and Adenosine triphosphate had higher affinity within biomarkers, which were the potential candidate components for treating BPH with Shenhailong formula, supported by their strong binding affinity with the protein of biomarkers. This study revealed the potential targets and potential candidate components of Shenhailong formula in the treatment of BPH, and confirmed that they had strong affinity. It provided a theoretical basis for the treatment of BPH with Shenhailong formula.

## 1. Introduction

Benign prostatic hyperplasia (BPH) is common among middle aged and elderly men, with the incidence gradually increasing with age.^[1]^ Epidemiological survey results show that the prevalence of BPH is approximately 50% to 75% in men over 50 years old and around 80% in men over 70 years old. The overall annual incidence ranges from 8.5/1000 to 41/1000.^[2]^ BPH is characterized by bladder outlet obstruction caused by hyperplasia of the prostatic stromal and glandular tissues, resulting in symptoms such as dysuria and a range of lower urinary tract symptoms including increased frequency, urgency, and pain.^[3]^ Lower urinary tract symptoms can lead to bladder wall thickening and bladder dysfunction, which result in acute urinary retention, urinary tract infection, bladder stones, and eventually lead to renal insufficiency.^[4]^ Therefore, BPH significantly diminishes patients’ quality of life and exerts a profound impact on their physical and mental health.^[5,6]^ The treatment methods for improving the clinical symptoms of BPH mainly include drug therapy, traditional surgery, and endovascular minimally invasive treatment. These treatment programs can help patients achieve varying degrees of improvement.^[7–9]^ However, some patients may experience a series of symptoms such as stress urinary incontinence, sexual dysfunction, secondary vesiculation in prostatic urethra wound, hematuria, and poor urination due to traditional surgery and minimally invasive treatment.^[10,11]^ Therefore, for most patients, safer drug therapy is a better choice, which includes α-receptor blockers, 5α-reductase inhibitors, M receptor antagonists, β-3 receptor agonists, and phosphodiesterase type 5 inhibitors.^[9,12,13]^ However, single western medicine treatment may also exhibit poor efficacy or lead to easy relapse.^[14–16]^ Therefore, some patients choose traditional Chinese medicine treatments or a combination of traditional Chinese and western medicine. Currently, certain Chinese patent medicines have shown promising treatment effects for BPH.^[17,18]^ Additionally, some scholars have explored the mechanism of traditional Chinese medicine prescriptions in the treatment of BPH.^[19,20]^ Consequently, the quest for new compositions of traditional Chinese medicine and new therapeutic targets will offer novel options for the prevention and treatment of BPH.

The Shenhailong formula is a confidential prescription from the family of Wang Chun-puk, a renowned doctor in Jiaodong. In the clinical practice of traditional Chinese medicine, it is used to treat weakness in the waist and knees, cold limbs, fatigue; dizziness and tinnitus, palpitations and insomnia, frequency of urination; sexual dysfunction caused by insufficient kidney Yang or BPH. It consists of 26 types of Chinese traditional medicine, such as Haima (*Hippocampus*), Wuweizi (*Schisandrae Chinensis Fructus*), Renshen (*Ginseng Radix et Rhizoma*), Fuling (*Poria*), Huangqi (*Astragali Radix*), Tiandong (*Asparagi Radix*), etc. Studies have confirmed that the most important function of Haima in traditional Chinese medicine is tonifying kidney yang, and it can be used for the treatment of BPH, erectile dysfunction, tumors, aging, inflammation, hypertension, and other conditions.^[21]^ The mature fruits of Wuweizi exhibit an antagonistic effect on phenylephrine-induced contractions, and they relax prostatic muscle strips through a noncompetitive antagonism mechanism. Its active compound, schisandrin A, can be used as a lead compound for the treatment of BPH.^[22]^ In addition, Piao et al found that Renshen can alleviate BPH symptoms while reducing the side effects caused by finasteride.^[23]^ Its function is to “Wenbushenyang Bushentianjing,” which translates to “warming and tonifying kidney Yang, supplementing marrow and filling essence.” In the clinical practice of traditional Chinese medicine, it is used to treat weakness in the waist and knees, cold limbs, fatigue; dizziness and tinnitus, palpitations and insomnia, frequency of urination; sexual dysfunction caused by insufficient kidney Yang or BPH. The therapeutic efficacy of this prescription for BPH is remarkable. Nevertheless, its underlying mechanisms of action remain elusive. Therefore, the exploration of biomarkers associated with the active components of Shenhailong formula in BPH can offer a novel theoretical foundation for the treatment of BPH using Shenhailong formula. However, although the abovementioned individual active ingredients have shown potential in the treatment of BPH, the overall efficacy and underlying mechanism of action of the Shenhailong formula remain unclear and require further research to clarify.

Mendelian randomization (MR) is a randomized approach based on Mendelian’s Second law, which uses genetic variation to test and estimate the causal effect of exposure factors on outcome, thereby avoiding the bias caused by reverse causality and confounding, where the exposure factors are those for which the researcher explores the causal relationship between them and the outcome.^[24]^ Single nucleotide polymorphisms (SNPs) are the most common instrumental variables (IVs) in MR studies.^[24]^ If the genetic variation related to the function or expression of the drug target protein can be used as an IV to study the factors interfering with the drug target, MR can be employed in drug development to investigate the possible efficacy and safety of drugs, as well as to explore the reuse potential and adverse reactions of existing drugs.^[25]^ Therefore, in this study, MR was used to screen biomarkers related to BPH.

This study was based on the transcriptome related data of BPH in the Gene Expression Omnibus database and the predicted target genes of the active ingredients of Shenhailong formula in the traditional Chinese medicine systems pharmacology (TCMSP) database. Through differential analysis, functional enrichment analysis, protein–protein interaction (PPI) network construction, MR analysis, and expression verification, 3 biomarkers were identified as related to the treatment of BPH. Thereafter, functional enrichment analysis, regulatory network construction, and molecular docking were performed, providing a new reference for exploring the molecular mechanism of BPH treatment by targeting biomarkers of the active ingredients of Shenhailong formula.

## 2. Materials and methods

### 2.1. Data source

The expression data of GSE7307 and GSE132714, which were related to BPH, were explored from the Gene Expression Omnibus database (https://www.ncbi.nlm.nih.gov/geo/). In GSE7307 (GPL570) dataset, 7 patients with BPH and 12 control samples (prostate tissues) were selected as the training set.^[26]^ The data of 18 BPH patients and 4 control samples (prostate tissues) in GSE132714 dataset were noted as the validation set.^[27]^

From Integrative Epidemiology Unit open genome-wide association study (GWAS) database (https://gwas.mrcieu.ac.uk/), the expression quantitative trait locus data of candidate genes was mined as the exposure factor.^[28]^ Searching for “Hyperplasia of prostate” in this database, an outcome variable dataset (ukb-b-8072) was retrieved, with 98,51,867 SNPs in 4133 BPH patients and 4,58,877 control samples of European populations. Samples of European origin and datasets with a relatively large sample size were selected as the data for this study. Explored the causal relationship between the exposure factor and the outcome. The data acquisition time was December 30, 2023.

### 2.2. Searching of target genes

From the ETCM database (http://www.tcmip.cn/ETCM/),^[29]^ the Chinese herbs included in the prescription of Shenhailong formula were found, that is, *Syngnathus*, *Hippocampus*, *Cervi Cornu Pantotrichum*, *Capra hircus Penis et Testis*, *Cnidii Fructus*, *Epimedii Folium*, *Cistanches Herba*, *Schisandrae Chinensis Fructus*, *Ginseng Radix et Rhizoma*, *Astragali Radix*, *Jujubae Fructus*, *Poria*, *Amomi Fructus*, *Dioscoreae Rhizoma*, *Zingiberis Rhizoma*, *Aconiti Lateralis Radix Praeparata*, *Angelicae Sinensis Radix*, *Rehmanniae Radix*, *Asparagi Radix*, *Ophiopogonis Radix*, *Lycii Fructus*, *Persicae Semen, Hirudo*, *Moutan Cortex*, *Achyranthis Bidentatae Radix*, and *Glycyrrhizae Radix et Rhizoma*. In the TCMSP database (https://www.91tcmsp.com/#/home),^[30]^ a total of 1865 active ingredients corresponding to Chinese herbs were retrieved, and in the HERB database (http://herb.ac.cn/),^[31]^ a total of 1198 active ingredients associated with Chinese herbs were obtained. Additionally, by querying the ETCM database (http://www.tcmip.cn/ETCM/) for relevant active ingredients of Chinese herbal medicine, a comprehensive collection of 3302 distinct compounds was acquired. The target genes of these active ingredients were identified using the TCMSP, HERB, Swiss Target Prediction (http://www.swisstargetprediction.ch/),^[32]^ and ETCM database, resulting in 19,671, 27,743, 8071 and 1252 target genes, respectively. Finally, 2879 Chinese herbs related target genes were obtained after merging. The target genes were explored by searching keywords “Benign prostatic hyperplasia” in the GeneCards database (https://www.genecards.org/)^[33]^ and DisGeNET database (https://www.disgenet.org/home/).^[34]^ A total of 779 BPH-related target genes were identified by merging the genes obtained from both databases.

### 2.3. Differential analysis

The gene expression matrix of training set was analyzed using the R package limma (V 3.54.0)^[35]^ to identify BPH-differentially expressed genes between BPH and control groups. The condition for screening was set as |log_2_ FoldChange| > 0.5 and *P*.adj value < .05. The volcano map was created by ggplot2 package (V 3.4.4),^[35]^ and heat map was drawn employing the pheatmap package (V 1.1.9).^[36]^ Whereafter, the ggvenn package (V 0.1.9)^[37]^ was implemented for the intersection of Chinese herbs related target genes, BPH-related target genes, and BPH-differentially expression genes to obtain candidate genes and draw venn diagram.

### 2.4. Function enrichment analysis and networks construction of candidate genes

To reveal the muti-component and muti-target synergistic characteristics of Shenhailong formular in exerting its effects, the Cytoscape software was utilized for the construction of a network visualizing the relationship between active ingredients and candidate genes (V 3.7.1).^[38]^ Soon after, the gene ontology and Kyoto Encyclopedia of Genes and Genomes enrichment analyses of candidate genes were performed to explore its related biological functions and pathways utilizing the cluster Profiler package (V 4.7.1.003; *P < *.05).^[39]^ The protein interaction information from the STRING database (https://string-db.org/) was utilized to acquire the interaction of candidate genes at protein level, with a medium confidence threshold set at 0.4.^[40]^ The isolated genes were excluded, and the PPI network was visualized using Cytoscape. Meanwhile, the Degree algorithm in Cytoscape was employed to rank the proteins and examine their connectivity.

### 2.5. MR analysis

To explore the causal relationship between candidate genes and BPH, we conducted MR analysis. It is of utmost importance to ensure that the following 3 conditions were met in MR studies: a significant correlation between IVs and exposure factors must be established, IVs should remain unaffected by confounding factors, and any influence of IVs on the outcome should solely occur through exposure factors.

The function extract instruments in the R package TwoSampleMR (V 0.5.6)^[41]^ were utilized to perform data reads of exposure factors and screening of IVs, with a threshold set at 5 × 10^−6^. To eliminate linkage disequilibrium, the threshold for that within 10 kb was set as an *R*^2^ value <0.001 (clump = TRUE). Meanwhile, the *F* statistics for IVs were calculated, and the IVs were weak and should be eliminated when *F* < 10 (*F* = (N − *k* − 1)/*k* × (*R*^2^/1 − *R*^2^), *R*^2^ represents the strength of the correlation between the IVs and the exposure factor. N is the sample size, and *k* denotes the number of IVs). Then based on the GWAS data of the outcomes and the IVs after the previous screening, the IVs that were significantly related to the outcomes were removed, thereby the IVs associated with exposure factors (candidate genes) that were not associated with outcome (BPH) were screened out (SNP ≥ 3). The effect alleles and effect sizes were unified by the function harmonize data, and the exposure factors-IVs-outcome matching was performed, missing values were filled in by interpolation. Moreover, MR analysis of candidate genes and BPH was conducted by 5 methods, namely MR-Egger,^[42]^ weighted median,^[43]^ inverse variance weighted (IVW),^[44]^ simple mode,^[44]^ weighted mode,^[45]^ wherein the results obtained from IVW served as the main reference (*P* < .05). The weighting formula for IVW was calculated as follows:


$$\begin{array}{l} W_{i}=\frac{1}{se^{2}\left({\hat{\;}\theta \;}_{i}\right)}\times \frac{\beta_{i}^{2}}{se^{2}\left(\gamma_{i}\right)} \end{array}$$

For the *i*th IV, β_*i*_ denoted the estimate of the effect of the IV on the exposure factor, γ_*i*_ denoted the estimate of the effect of the IV on the outcome, and se denoted the standard error. If there were fewer missing values, the observations containing the missing values were deleted directly, and the na.omit function could be used to remove the missing values. If there were more missing values, multiple interpolation would be used to fill in the missing values, with mice package. The results were visually depicted through the utilization of scatter plots, forest plots, and funnel plots. An odds ratio above 1 indicated a risk factor for BPH, while a value below 1 suggested its potential as a protective factor.

### 2.6. The sensitivity analysis

The reliability of MR analysis was evaluated by sensitivity analysis, consisting of heterogeneity test, horizontal pleiotropy test, and leave-one-out analysis. Specifically, to investigate the effect of heterogeneity, we performed heterogeneity analysis by using the R package “TwoSampleMR” function mr_heterogeneity (*P* > .05). And the presence of confounding factors in this study was assessed by conducting horizontal pleiotropy test using the mr_pleiotropy_ function (*P* > .05). At the same time, the mr_leaveoneout function was employed to conduct leave-one-out analysis, aiming to evaluate the potential impact of an individual SNP on the overall outcomes. And the candidate genes screened by the above analysis were identified as potential biomarkers.

### 2.7. Recognition of biomarkers

The potential biomarkers obtained by MR were used for expression verification in the training set, and the genes that were consistent with the MR results were selected for further verification in the validation set. After that, genes whose expression trend in the validation set was consistent and significant with that in the training set were noted as biomarkers.

### 2.8. Gene set enrichment analysis (GSEA) of biomarkers

Based on the median value of biomarkers expression levels, samples within the trainings set were divided into high and low expression groups, and the R package clusterProfiler (V 4.7.1.003)^[46]^ was utilized to conduct gene set enrichment analysis (GSEA) on biomarkers (*P* < .05). The hallmark enrichment analysis pathway set (org.Hs.e.g.db) was obtained from the Molecular Signatures Database (https://www.gsea-msigdb.org/gsea/msigdb) as the annotated gene set. The enriched pathways were ranked using false discovery rate and normalized enrichment score.

### 2.9. Construction of networks and molecular docking

The mirTARbase (https://awi.cuhk.edu.cn/~miRTarBase) and NetworkAnalyst (https://www.networkanalyst.ca/NetworkAnalyst/) databases were separately utilized for the prediction of miRNAs and transcription factors (TFs) associated with biomarkers. Finally, Cytoscape software was used for visual of TF-mRNA-miRNA regulatory network. In the meantime, the Cytoscape software was also employed to construct the network of active ingredient-biomarker interactions, whereby active ingredients targeting biomarkers were identified as pivotal compounds. The accuracy of network pharmacological prediction was verified by conducting molecular docking to confirm the interaction between compounds and biomarkers in the treatment of BPH. The protein 3-dimensional structure of biomarkers was downloaded from the Research Collaboratory for Structural Bioinformatics database (https://www.rcsb.org/),^[47]^ while the MOL2 structure of the pivotal compounds was obtained from the TCMSP database (https://www.91tcmsp.com/#/home). SYBYL-X software (V 2.1.1)^[48]^ was utilized for molecular docking, enabling the binding activity determination between pivotal compounds (ligands) and biomarkers (receptors), and the PyMOL software (https://pymol.org) was applied for the visualization. A higher total score indicates a higher affinity of the receptor to the ligand.

### 2.10. Statistical analysis

The R software (V 4.2.2) was utilized for processing and analyzing the data. A significance level of *P* < .05 was considered to indicate statistical significance.

## 3. Results

### 3.1. Acquisition of 41 candidate genes

**Figure 1.**
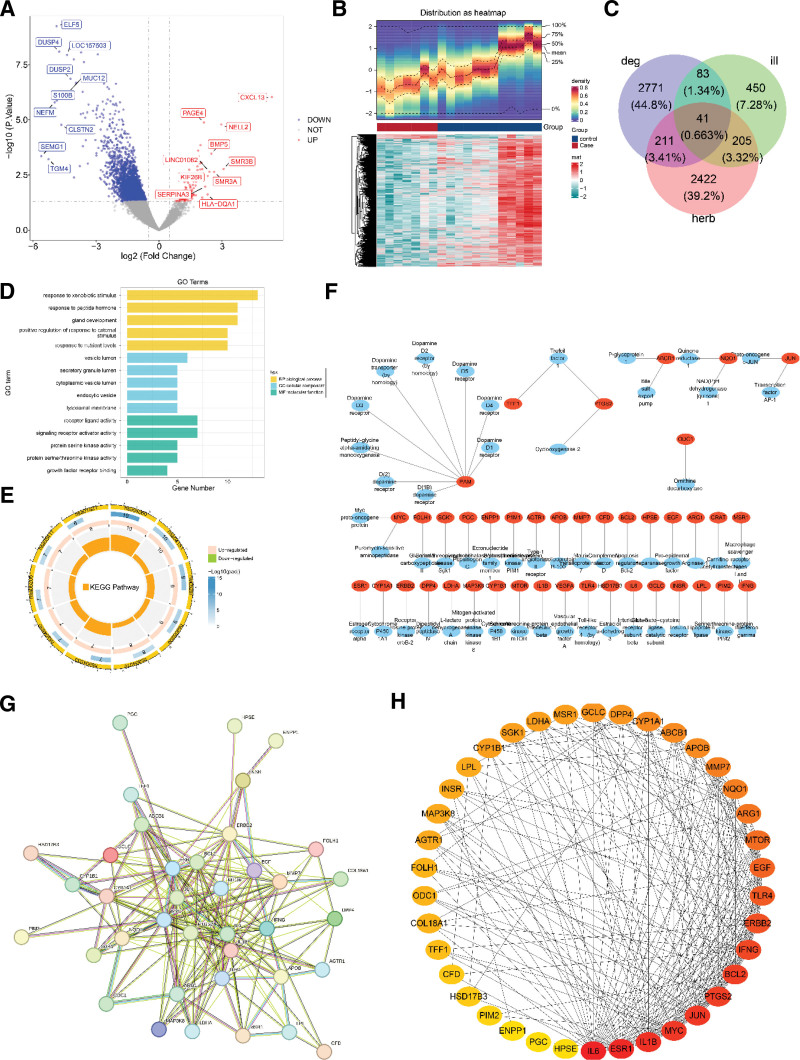
Acquisition of candidate genes. (A, B) The volcano map (labelled genes are differentiation multiples |log_2_ FC| maximum top10) and heat map of BPH-DEGs in GSE7307. (C) Candidate genes obtained in the intersection of Chinese herb-related target genes (herb), BPH-related target genes (ill), and BPH-DEGs (deg). (D) GO functional enrichment analysis of the candidate genes. (E) KEGG pathways in which the candidate genes enriched. (F) The active ingredient-candidate gene network (genes in red, corresponding compounds in blue). (G, H) PPI network of the candidate genes. BPH = benign prostatic hyperplasia, DEGs = differentially expressed genes, GO = gene ontology, KEGG = Kyoto Encyclopedia of Genes and Genomes, PPI = protein–protein interaction.

The difference analysis of GSE7307 identified a total of 3106 BPH-differentially expressed genes (DEGs), comprising 105 upregulated and 3001 downregulated BPH-DEGs (Fig. 1A, B). The intersection of 2879 Chinese herb-related target genes (herb), 779 BPH-related target genes (ill), and 3106 BPH-DEGs (deg) was undertaken, thereby obtaining of 41 candidate genes (Fig. 1C). Gene ontology functional enrichment analysis of the candidate genes obtained 1020 results, including 965 biological processes (BP, e.g., response to xenobiotic stimulus, response to peptide hormone), 32 cellular components (e.g., vesicle lumen, secretory granule lumen) and 23 molecular functions (e.g., receptor ligand activity, signaling receptor activator activity; Fig. 1D). The candidate genes were notably enriched in 101 Kyoto Encyclopedia of Genes and Genomes pathways, such as HIF-1 signaling pathway, PI3K-Akt signaling pathway, and Chemical carcinogenesis-receptor activation (Fig. 1E). The active ingredient-candidate gene network showed that PAM corresponded to 9 compounds, JUN, NQO1, PTQB2, ABCB1, and MYC corresponded to 2 compounds, the remaining genes corresponded to only 1 compound (Fig. 1F). Furthermore, there were 112 pairs of interactions in the PPI network. To be specific, IL6, ESP1, ILB, MYC, JUN, PTGS2, BCL2, and IFNG had high connectivity and interacted with multiple genes, while ectonucleotide pyrophosphatase/phosphodiesterase family member 1 (ENPP1), PGC, and HPSE had poor connectivity and only interacted with 1 gene (Fig. 1G, H).

### 3.2. Discovery of potential biomarkers associated with BPH

**Figure 2.**
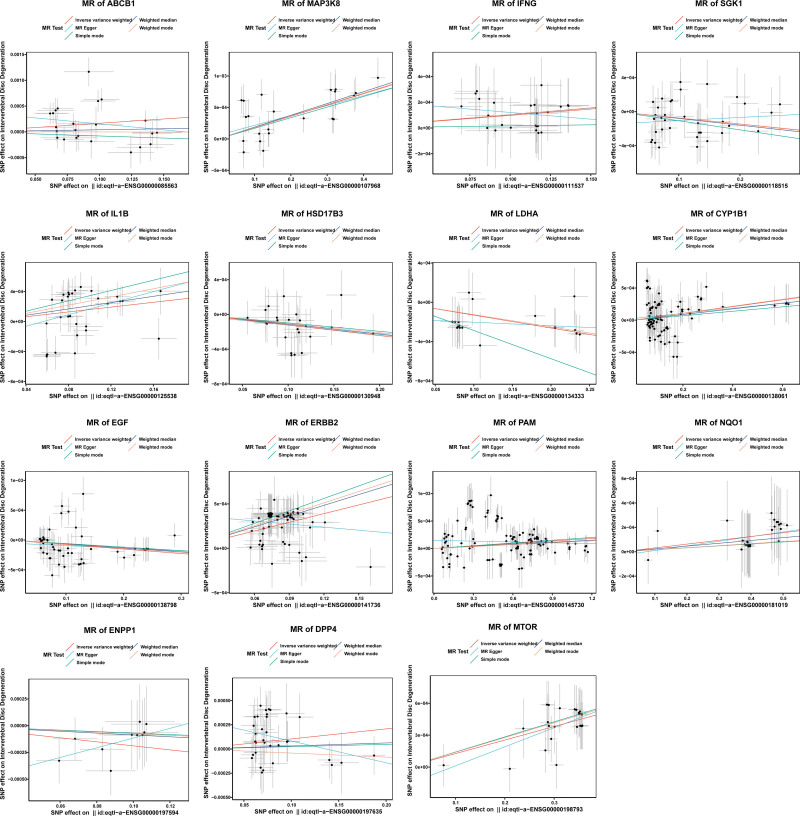
Scatter plot of the potential effects of candidate gene associated SNPs on BPH. BPH = Benign prostatic hyperplasia, ENPP1 = ectonucleotide pyrophosphatase/phosphodiesterase family member 1, MR = Mendelian randomization, SNP = single nucleotide polymorphism, SGK1 = serum/glucocorticoid-regulated kinase 1.

**Figure 3.**
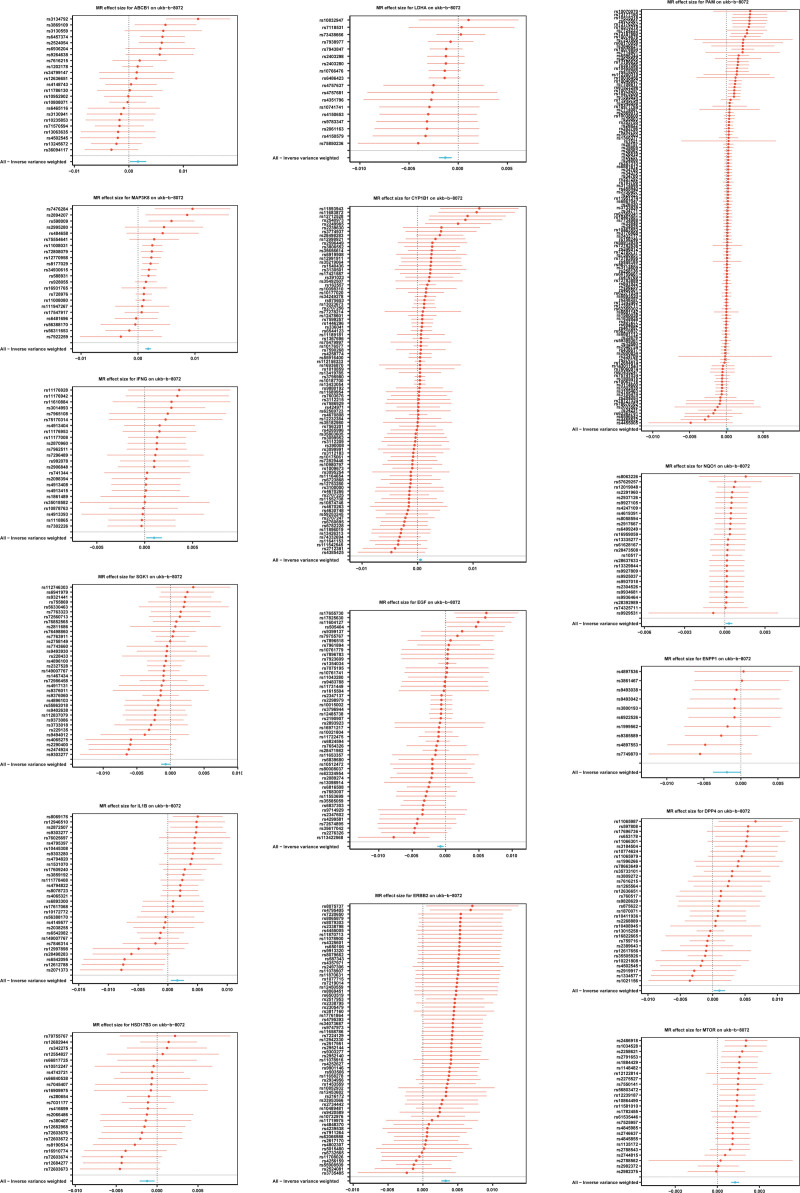
Forrest plot of the potential effects of the candidate gene associated SNP on BPH. BPH = benign prostatic hyperplasia, SNP = single nucleotide polymorphism.

**Figure 4.**
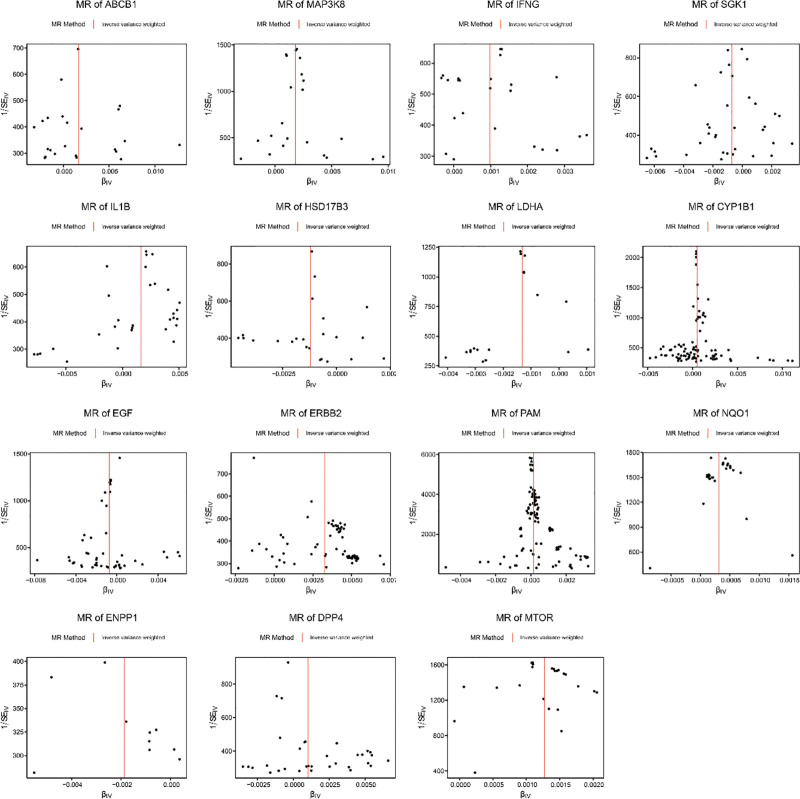
Funnel plot of the causal effect of the candidate gene related SNPs on BPH. BPH = benign prostatic hyperplasia, EGF = epidermal growth factor, ENPP1 = ectonucleotide pyrophosphatase/phosphodiesterase family member 1, MR = Mendelian randomization, SNP = single nucleotide polymorphism, SGK1 = serum/glucocorticoid-regulated kinase 1.

A total of 15 genes were identified through MR analysis, which was conducted on candidate gene of BPH. Among them, ERBB2, MAP3K8, ABCB1, IL1B, MTOR, DPP4, IFNG, CYP1B1, NQO1, PAM were risk factors (odds ratio > 1), while serum/glucocorticoid-regulated kinase 1 (SGK1), epidermal growth factor (EGF), HSD17B3, LDHA, ENPP1 were protective factors (odds ratio < 1) for BPH (Supplementary Material 1, Supplemental Digital Content, https://links.lww.com/MD/Q552). In the scatter plot, there was a positive correlation between the effects of SNPs on risk factors for BPH overall, while the correlation of protective factors was negative (Fig. 2). Meanwhile, the MR effect size of risk factors exceeded zero, and that of protective factors were less than zero in the forest map (Fig. 3). These findings validate the aforementioned conclusions derived from the MR analysis. Finally, the funnel plot indicated that the MR analysis was consistent with Mendel’s second law of random assortment (Fig. 4).

The horizontal pleiotropy test indicated that *P* values of ERBB2, PAM, and DPP4 were <.05 and were affected by confounding factors (Supplementary Material 2, Supplemental Digital Content, https://links.lww.com/MD/Q552). The heterogeneity test revealed that the *P* values of ABCB1, IL1B, and PAM were <.05. However, significant results of them were obtained in IVW, indicating that the results were not affected by heterogeneity (Supplementary Material 3, Supplemental Digital Content, https://links.lww.com/MD/Q552). Sensitivity analysis showed that the MR analysis of the 12 genes (ABCB1, MAP3K8, IFNG, SGK1, IL1B, HSD17B3, LDHA, CYP1B1, EGF, NQO1, ENPP1, MTOR) were reliable, and they were potential biomarkers for BPH (Supplementary Material 4, Supplemental Digital Content, https://links.lww.com/MD/Q553).

### 3.3. SGK1, EGF, and ENPP1were biomarkers for BPH

Five protective factors, SGK1, HSD17B3, LDHA, EGF and ENPP1, were all downregulated in the training set, which was consistent with the results of MR (Supplementary Material 5, Supplemental Digital Content, https://links.lww.com/MD/Q553). Their expression levels were further verified in the validation set, and SGK1, EGF, and ENPP1 showed significant and consistent expression trends with those in the training set, thus serving as biomarkers (Supplementary Material 6, Supplemental Digital Content, https://links.lww.com/MD/Q553). Acquisition of biomarkers provided a new target for treatment of BPH.

### 3.4. All biomarkers were involved in kras signaling dn, and pancreas beta cells, etc

**Figure 5.**
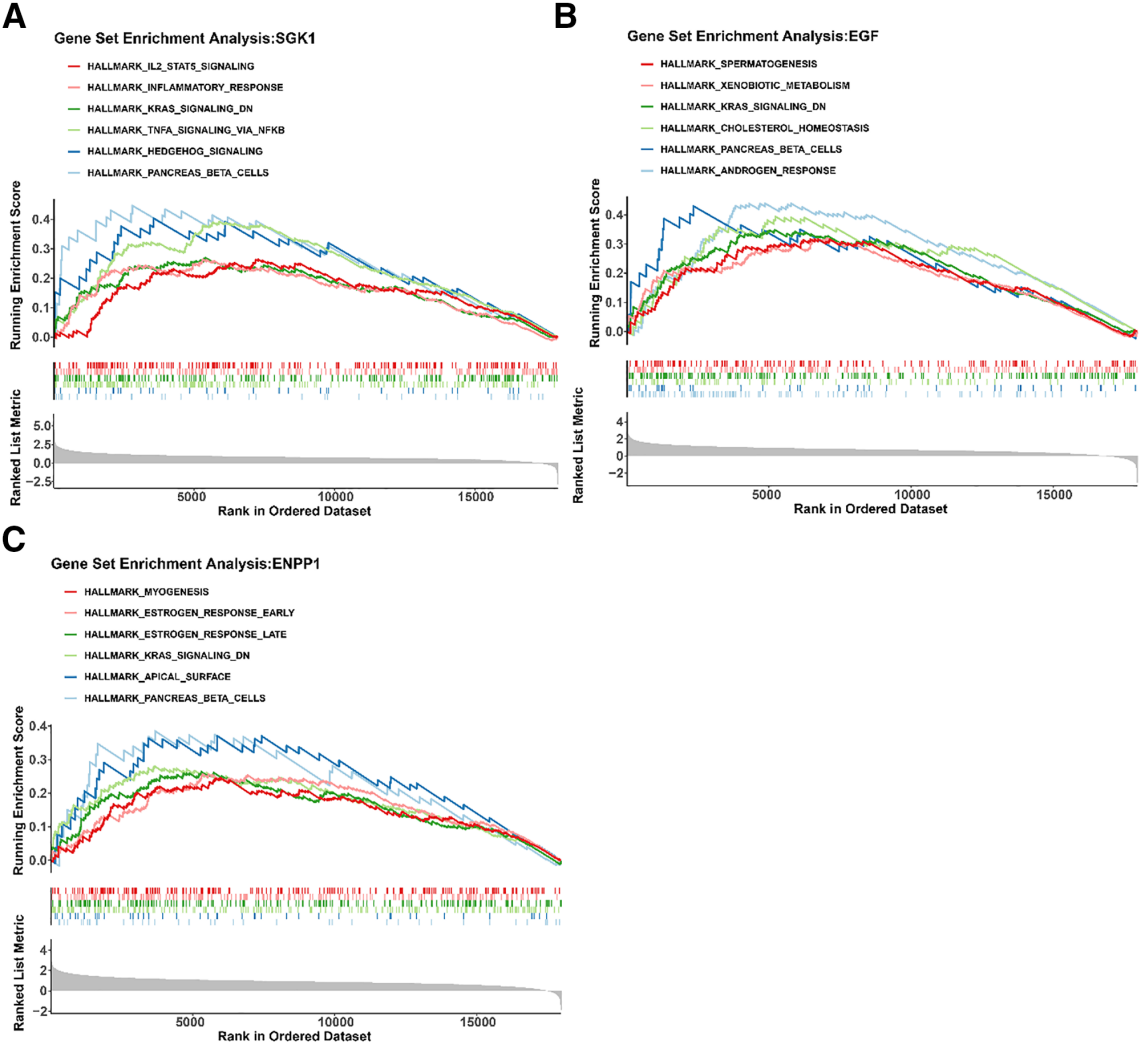
GSEA of SGK1, EGF and ENPP1. (A) SGK1, (B) EGF, and (C) ENPP1. EGF = epidermal growth factor, ENPP1 = ectonucleotide pyrophosphatase/phosphodiesterase family member 1, GSEA = gene set enrichment analysis, SGK1 = serum/glucocorticoid-regulated kinase 1.

The GSEA analysis examined the biological pathways associated with the biomarkers. The pathways enriched by SGK1 were pancreas beta cells, hedgehog signaling, and tnfa signaling via nfkb, etc (Fig. 5A). EGF was highly expressed in androgen response, pancreatic beta cells, cholesterol homeostasis and other pathways (Fig. 5B). ENPP1 was notably enriched in myogenesis, estrogen response early, and estrogen response late, etc (Fig. 5C). All 3 biomarkers (SGK1, EGF, and ENPP1) were significantly enriched in the “kras signaling dn,” “pancreas beta cells,” “estrogen response early,” “estrogen response late,” and “spermatogenesis” pathways (GSEA, false discovery rate < 0.05).” These pathway enrichment results provided evidence at the pathway level for elucidating how Shenhailong formula exerts its therapeutic effects by regulating these biomarkers and related pathways.

### 3.5. The relationship between TFs and miRNAs regulation of biomarkers was complex

There were 29 TFs and 101 microRNAs were predicted for the construction of TF-mRNA-miRNA network (Supplementary Material 7, Supplemental Digital Content, https://links.lww.com/MD/Q553). In detail, GATA2 and JUN exerted their influence on 3 biomarkers, and the actions of hsa-miR-4459 and hsa-miR-4433a-3p were involved in the regulation of EGF and ENPP1. These TFs and miRNAs might play crucial roles in the regulatory mechanisms governing the expression of biomarkers.

### 3.6. Stepholidine and Adenosine triphosphate were the potential candidate components for treating BPH with Shenhailong formula

**Figure 6.**
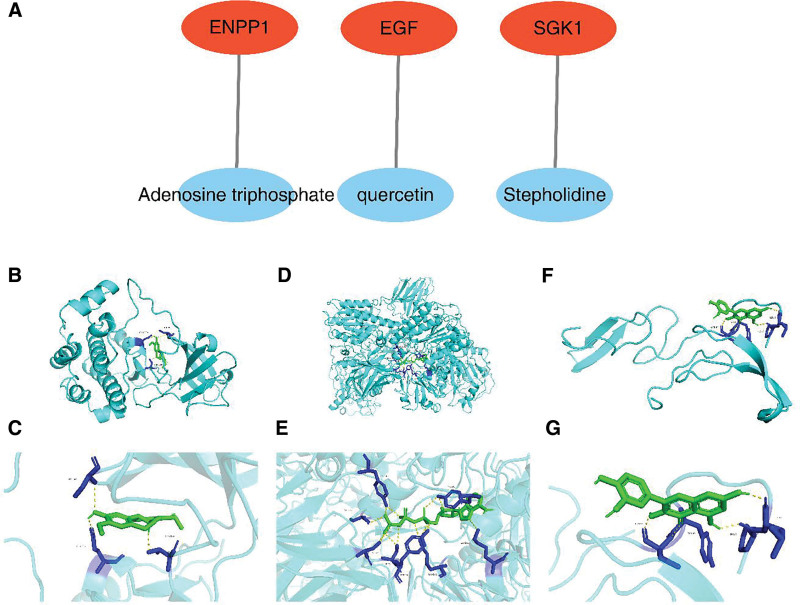
Active ingredient-biomarker network and molecular docking. (A) Active ingredient-biomarkers network, biomarkers in red and corresponding compounds in blue. (B, C) Molecular docking of SGK1 and stepholidine. (D, E) Molecular docking of ENPP1 and adenosine triphosphate. (F, G) Molecular docking of EGF and quercetin. EGF = epidermal growth factor, ENPP1 = ectonucleotide pyrophosphatase/phosphodiesterase family member 1.

In the active ingredient-biomarker network, Stepholidine modulated SGK1, quercetin influenced EGF, and Adenosine triphosphate acted on ENPP1 (Fig. 6A). The total score in molecular docking of SGK1 and Stepholidine was −9.4, while that of ENPP1 and Adenosine triphosphate was −9.7, indicating a strong binding affinity between the compounds and the proteins (Figs. 6B–G). Therefore, Stepholidine and Adenosine triphosphate were the potential candidate components for treating BPH with Shenhailong formula.

## 4. Discussion

BPH is a prevalent disease in middle aged and elderly men, which has a significant impact on the quality of life of patients.^[46,47]^ Traditional Chinese medicine is an effective means to treat BPH, but its effective components and mechanism are still not clear.

Shenhailong formula could warm and tonify kidney yang, nourish marrow and fill essence. In this prescription, Hailong is the sovereign drug, and is combined with minister drugs such as Haima and other drugs to absorb essence and stop the stagnation, Renshen and other drugs to replenish qi and spleen, Fuzi and other drugs to warm middle-Jiao and promote yang, Danggui and other drugs to nourish blood and produce fluid. In addition, Taohe and other drugs are used as assistant drugs to promote blood circulation and regulate blood, and Gancao is used as conductant drug to reconcile various drugs. Shenhailong formula has been proven to have outstanding clinical efficacy, but its effective components and mechanism remain unclear. In this study, MR analysis and network pharmacology were used to study its possible effective components and mechanism. And a series of signaling pathways, TFs, miRNA, protein and chemical components, which play important roles in the treatment of BPH, was identified. These findings may lay a foundation for further explanation of the effective components and mechanism.

MR studies of the protective factors of BPH SGK1, EGF and ENPP1 in GSE7307 and GSE132714 showed a consistent trend of expression significantly downregulated in the disease group, which could be used as biomarkers for Shenhailong formula treating BPH. All biomarkers showed enrichment in kras signaling dn, pancreatic beta cells, early estrogen response, late estrogen response, and spermatogenesis. In addition, TFs GATA2 and JUN had effects on all 3 biomarkers, and hsa-miR-4459 and hsa-miR-4433a-3p were involved in the regulation of EGF and ENPP1. Stepholidine and Adenosine triphosphate are the main active ingredients in the treatment of BPH with Shenhailong formula, and they have strong binding ability with biomarker proteins.

This study found that 3 biomarkers, SGK1, EGF and ENPP1, are closely related to the treatment of BPH by Shenhailong formula. EGF was the prototype of the type I EGF family, can combine with EGFR and activate its intrinsic tyrosine kinase activity, and then start the signal transduction pathway, regulate a variety of cells, especially the epithelial cell proliferation, differentiation, and migration.^[49,50]^ EGF has been implicated in the development of BPH.^[51,52]^ EGF can induce the proliferation of human prostate stromal cells reaction,^[53]^ the EGF and EGFR expression in BPH prostate tissue, and may play a role in the form of autocrine, at the same time, the EGF gene SNP is associated with BPH prostate volume and concentration of PSA.^[54]^ In addition, EGF acts in concert with sex hormones and plays an important role in the pathological process of BPH. The production of EGF is regulated by androgens and the proliferation response of epithelial cells to androgens also requires the participation of EGF.^[55]^ The α1 adrenoceptor mediated human hyperplastic prostate cells proliferation is impaired by EGF receptor inhibition.^[56]^ In recurrent prostate cancer^[57]^ and bladder cancer cells,^[58]^ EGF can activate the AR signaling pathway and promote the proliferation of tumor cells. EGF can also reduce the expression of androgen receptor (AR) in differentiated mouse vas deferens epithelial cells, inhibit the signal transduction of androgen signaling, and promote the reentry of differentiated cells into the cell cycle.^[59]^ These results suggest that EGF and its interaction with the androgen signaling pathway are important targets for the treatment of BPH. In this study, EGF is the main biomarker for the treatment of BPH and is enriched in androgen response signaling pathways, suggesting that EGF may play a role in the treatment of BPH by regulating the expression of EGF and acting on the androgen signaling pathway. Indeed, several studies have shown that this pathway is an important mechanism for the treatment of BPH drugs. Angelica and Astragalus decoction can reduce the levels of serum EGF and DHT and downregulate the expression of AR and 5α-reductase in BPH model rats.^[60]^ Qianliening capsule reducing the expression of EGF and EGFR in BPH rats, and further inhibition of STAT3 mediated Bcl-2, Cyclin D1 expression.^[61]^ *Abacopteris penangiana* total flavanosine and its acid hydrolysate decreased EGF and DHT contents, inhibited Bcl-2 expression and increased Bax and p53 expression in BPH rats.^[62]^ Canagliflozin reduces BPH and prostate EGF and EGFR and rat serum DHT AR expression, reduce the STAT phosphorylation and proliferation related protein expression.^[63]^

SGK1 (serine/threonine kinase) as a key member of the AGC kinase family, plays a core role in integrating intracellular and extracellular signals to regulate cell survival, proliferation, apoptosis, ion homeostasis, and stress responses.^[64,65]^ The core pathological features of BPH are excessive cell proliferation and suppressed apoptosis, a process coordinately regulated by the PI3K/Akt/mTOR signaling pathway.^[66–68]^ As a downstream effector molecule of the PI3K pathway, SGK1 can be phosphorylated and activated by PDK1 and mTORC2, thereby potentially driving abnormal proliferation of prostate cells through multiple mechanisms.^[69]^ Meanwhile, through RNA-Seq analysis, Xiao et al found that 6 anti-apoptotic genes, including SGK1, are involved in HJZ-12-induced BPH cell death and play a significant role in inhibiting BPH progression.^[70]^ In addition, chronic low-grade inflammation is a critical driver of BPH progression. As a key regulator of inflammatory signaling, SGK1 may exacerbate this process by activating the NF-κB pathway, promoting the nuclear translocation of NF-κB, and enhancing the transcription of pro-inflammatory cytokines, thereby facilitating the development of BPH.^[71,72]^ The results of this study further confirm that SGK1 is significantly enriched in the TNF-α-mediated NF-κB signaling pathway, reinforcing its critical role in this pathway. In summary, SGK1 may play an important role in the occurrence and development of BPH by participating in the PI3K/Akt/mTOR pathway to regulate cell proliferation and apoptosis, as well as activating the NF-κB pathway to associate with inflammation. These findings are expected to provide new theoretical basis for mechanism research and targeted therapy of BPH.

ENPP1 is a widely expressed metalloenzyme whose core function is to catalyze the hydrolysis of nucleotides (e.g., ATP, GTP), generating nucleoside monophosphates (AMP, GMP) and inorganic pyrophosphate.^[63]^ This process not only regulates the homeostasis of extracellular purinergic signaling but also participates in various physiological and pathological processes such as mineralization balance and immune activation (PMID: 30799235).^[73]^ In recent years, it has been found that ENPP1 plays an important regulatory role in the STING signaling pathway by hydrolyzing cGAMP, which has attracted more and more attention in immune regulation.^[74]^ As a key pathway in innate immunity, the STING pathway can activate downstream inflammatory responses and promote the release of pro-inflammatory cytokines (e.g., IFN-β, TNF-α), thereby participating in the maintenance of immune homeostasis and the regulation of inflammatory processes.^[75,76]^ Chronic inflammation, which leads to tissue damage and the release of pro-inflammatory cytokines, plays an important role in the pathogenesis of BPH.^[77]^ However, based on its regulatory function in STING-mediated inflammatory signaling, it is hypothesized that ENPP1 may be involved in the pathological process of BPH by modulating the local immune microenvironment of the prostate. This association awaits further research for verification.

According to the results of this study, SGK1 and ENPP1 are the biomarkers of Shenhailong formula in the treatment of BPH, and are enriched in multiple pathways, such as KRAS_SIGNALING_DN, ESTROGEN_RESPONSE_EARLY, and ESTROGEN_RESPONSE_LATE. It is suggested that they may be new targets and mechanisms for the treatment of BPH, which are worthy of further study.

Molecular docking results show that Stepholidine and Adenosine triphosphate are of great value in the treatment of BPH by Shenhailong formula, and they have high binding activities with SGK1 and ENPP1 respectively. Stepholidine has been reported to have dopamine receptor D1 agonist activity and dopamine receptor D2 antagonist activity, and has neuroprotective effects on methamphetamine-induced memory impairment in mice,^[78]^ and has been used in drug withdrawal studies.^[79]^ Adenosine triphosphate, as the most direct energy source in the body, is widely distributed. Studies have shown that ATP is reduced in the urothelium of BPH patients with detrusor underactivity,^[80]^ while ATP hydrolysis disorder reduces adenosine A1 receptor-mediated inhibition of cholinergic nerve activity in BPH patients with bladder outlet obstruction.^[81]^ However, there is no direct study of Stepholidine and Adenosine triphosphate applied in the treatment of BPH, and the findings of this study may provide some new chemical components and targets for the treatment of BPH.

In this study, network pharmacology and MR were used to study the mechanism of Shenhailong formula in the treatment of BPH, and a series of biomarkers, signaling pathways, regulatory networks and chemical components of Shenhailong formula in the treatment of BPH were obtained. The 2 biomarkers, SGK1 and ENPP1, and 2 chemical compounds, Stepholidine and Adenosine triphosphate, were first discovered playing a potential role in the treatment of BPH. These findings lay a good foundation for revealing the mechanism of action of Shenhailong formula in the treatment of BPH.

Although bioinformatics analysis has provided potential targets and candidate components for the treatment of BPH with the Shenhailong formula, this study still has certain limitations. First, due to the lack of biological experimental verification, the functions of the screened biomarkers have not been confirmed. Second, although the identified potential candidate component (Stepholidine) has been extensively studied in the field of neuroscience, its in vivo validation in the prostate-related field has not yet been reported. Third, MR relies on GWAS data from public databases, and the sample sources are mainly from European populations, which may lead to differences in population applicability.

In future studies, we will further verify the role of biomarkers by combining in vitro and in vivo experiments. We plan to conduct systematic pharmacokinetic and pharmacodynamic evaluations, and deeply explore the efficacy and dose-effect relationship of Stepholidine in the prostate through the construction of animal models of prostate-related diseases, so as to clarify its potential therapeutic value. In addition, more diverse populations will be included in the research to improve the generalizability of the results.

## 5. Conclusion

Through the integration of network pharmacology and MR analysis, the mechanism of Shenhailong formula in the treatment of BPH was studied, and a series of biomarkers, signaling pathways, regulatory networks and chemical components was revealed.

The 3 biomarkers, SGK1, EGF and ENPP1, and 2 chemical compounds, Stepholidine and Adenosine triphosphate were highlighted to play an important role in the treatment of BPH. These findings may offer novel perspectives on the mechanism underlying Shenhailong formula in BPH treatment.

## Author contributions

**Conceptualization:** Jia Pan, Mengyao Yu, Lihan Zhang, Jing Yin, Peihai Zhang, Xiaoyuan Li.

**Data curation:** Jia Pan, Mengyao Yu, Lihan Zhang, Jing Yin, Shuwu Zhang, Peihai Zhang, Qingying Fan, Jiatong Liu, Xiaoyuan Li.

**Formal analysis:** Jia Pan, Mengyao Yu, Lihan Zhang, Jie Liu, Shuwu Zhang, Qingying Fan, Xiaoyuan Li.

**Funding acquisition:** Xiaoyuan Li.

**Investigation:** Jia Pan, Mengyao Yu, Lihan Zhang, Jing Yin, Jie Liu, Shuwu Zhang, Jiatong Liu.

**Methodology:** Jia Pan, Mengyao Yu, Lihan Zhang, Jing Yin, Jie Liu, Jiatong Liu, Xiaoyuan Li.

**Resources:** Qingying Fan.

**Software:** Shuwu Zhang.

**Supervision:** Jing Yin, Jie Liu, Shuwu Zhang, Qingying Fan, Xiaoyuan Li.

**Validation:** Jia Pan, Mengyao Yu, Lihan Zhang, Jing Yin, Jie Liu, Peihai Zhang, Qingying Fan, Xiaoyuan Li.

**Visualization:** Jia Pan, Mengyao Yu, Lihan Zhang, Jing Yin, Jie Liu, Peihai Zhang, Xiaoyuan Li.

**Writing – original draft:** Jia Pan, Mengyao Yu, Lihan Zhang, Jing Yin, Xiaoyuan Li.

**Writing – review & editing:** Jia Pan, Mengyao Yu, Lihan Zhang, Jing Yin, Xiaoyuan Li.

## Supplementary Material





## References

[1] Devlin CM Simms MS Maitland NJ. Benign prostatic hyperplasia - what do we know? BJU Int. 2021;127:389–99. 32893964 10.1111/bju.15229

[2] Egan KB. The epidemiology of benign prostatic hyperplasia associated with lower urinary tract symptoms: prevalence and incident rates. Urol Clin North Am. 2016;43:289–97. 27476122 10.1016/j.ucl.2016.04.001

[3] Launer BM McVary KT Ricke WA Lloyd GL. The rising worldwide impact of benign prostatic hyperplasia. BJU Int. 2021;127:722–8. 33124118 10.1111/bju.15286 PMC8170717

[4] Lerner LB McVary KT Barry MJ. Management of lower urinary tract symptoms attributed to benign prostatic hyperplasia: AUA GUIDELINE PART I-initial work-up and medical management. J Urol. 2021;206:806–17. 34384237 10.1097/JU.0000000000002183

[5] Zhu C Wang DQ Zi H. Epidemiological trends of urinary tract infections, urolithiasis and benign prostatic hyperplasia in 203 countries and territories from 1990 to 2019. Mil Med Res. 2021;8:64. 34879880 10.1186/s40779-021-00359-8 PMC8656041

[6] Foo KT. What is a disease? What is the disease clinical benign prostatic hyperplasia (BPH)? World J Urol. 2019;37:1293–6. 30805683 10.1007/s00345-019-02691-0 PMC6620380

[7] Miernik A Gratzke C. Current treatment for benign prostatic hyperplasia. Dtsch Arztebl Int. 2020;117:843–54. 33593479 10.3238/arztebl.2020.0843 PMC8021971

[8] Sciacqua LV Vanzulli A Di Meo R. Minimally invasive treatment in Benign Prostatic Hyperplasia (BPH). Technol Cancer Res Treat. 2023;22:22. 10.1177/15330338231155000 PMC9936536 36794408

[9] Plochocki A King B. Medical treatment of benign prostatic hyperplasia. Urol Clin North Am. 2022;49:231–8. 35428429 10.1016/j.ucl.2021.12.003

[10] Franco JVA Jung JH Imamura M. Minimally invasive treatments for benign prostatic hyperplasia: a Cochrane network meta-analysis. BJU Int. 2022;130:142–56. 34820997 10.1111/bju.15653

[11] Ottaiano N Shelton T Sanekommu G Benson CR. Surgical complications in the management of benign prostatic hyperplasia treatment. Curr Urol Rep. 2022;23:83–92. 35262855 10.1007/s11934-022-01091-z

[12] Ganesan V Agarwal D. Medical advancements in benign prostatic hyperplasia treatments. Curr Urol Rep. 2024;25:93–8. 38448685 10.1007/s11934-024-01199-4

[13] Koudonas A Anastasiadis A Tsiakaras S. Overview of current pharmacotherapeutic options in benign prostatic hyperplasia. Expert Opin Pharmacother. 2023;24:1609–22. 37448198 10.1080/14656566.2023.2237406

[14] Wang X Wang X Li S Meng Z Liu T Zhang X. Comparative effectiveness of oral drug therapies for lower urinary tract symptoms due to benign prostatic hyperplasia: a systematic review and network meta-analysis. PLoS One. 2014;9:e107593. 25216271 10.1371/journal.pone.0107593 PMC4162615

[15] Fan Z Shi H Zhang J Wang H Wang J. Comparative efficacy of different drugs for lower urinary tract symptoms due to benign prostatic hyperplasia: a bayesian network meta-analysis. Front Pharmacol. 2022;13:763184. 35330833 10.3389/fphar.2022.763184 PMC8940212

[16] Yoosuf BT Panda AK Kt MF Bharti SK Devana SK Bansal D. Comparative efficacy and safety of alpha-blockers as monotherapy for benign prostatic hyperplasia: a systematic review and network meta-analysis. Sci Rep. 2024;14:11116. 38750153 10.1038/s41598-024-61977-5 PMC11096304

[17] Li S Lu A Wang Y. Symptomatic comparison in efficacy on patients with benign prostatic hyperplasia treated with two therapeutic approaches. Complement Ther Med. 2010;18:21–7. 20178875 10.1016/j.ctim.2009.10.002 PMC7126207

[18] Zeng H Wang Z Zhu W. Comparative efficacy of commercial oral poly-herbal traditional Chinese medicine formulations combined with western medicine in benign prostatic hyperplasia management: a systematic review and network meta-analysis. Front Pharmacol. 2024;15:1358340. 38904002 10.3389/fphar.2024.1358340 PMC11187581

[19] Lihua J Haodan K Yuan XU. Efficacy of Buzhong Yiqi decoction on benign prostatic hyperplasia and its possible mechanism. J Tradit Chin Med. 2023;43:533–41. 37147755 10.19852/j.cnki.jtcm.2023.03.003 PMC10133955

[20] Shaofeng C Chunxu L Qiang G. The mechanism of Lingze tablets in the treatment of benign prostatic hyperplasia based on network pharmacology and molecular docking technology. Andrologia. 2022;54:e14555. 36064190 10.1111/and.14555

[21] Chen L Wang X Huang B. The genus Hippocampus--a review on traditional medicinal uses, chemical constituents and pharmacological properties. J Ethnopharmacol. 2015;162:104–11. 25560669 10.1016/j.jep.2014.12.032

[22] Wei F He X Xu K Wang S. Stepwise frontal analysis coupled with cell membrane chromatography for affinity screening and characterization analysis of bioactive constituent from the mature fruits of schisandra chinensis. J Chromatogr B Analyt Technol Biomed Life Sci. 2020;1161:122443. 10.1016/j.jchromb.2020.122443 33246280

[23] Park JY Park WY Song G. Panax ginseng C.A. meyer alleviates benign prostatic hyperplasia while preventing finasteride-induced side effects. Front Pharmacol. 2023;14:1039622. 36713838 10.3389/fphar.2023.1039622 PMC9877295

[24] Teng Y Huang DQ Li RX Yi C Zhan YQ. Association between telomere length and risk of lung cancer in an asian population: a mendelian randomization study. World J Oncol. 2023;14:277–84. 37560336 10.14740/wjon1624 PMC10409562

[25] Gill D Georgakis MK Walker VM. Mendelian randomization for studying the effects of perturbing drug targets. Wellcome Open Res. 2021;6:16. 33644404 10.12688/wellcomeopenres.16544.1 PMC7903200

[26] Zhai YJ Feng Y Ma X Ma F. Defensins: defenders of human reproductive health. Hum Reprod Update. 2023;29:126–54. 36130055 10.1093/humupd/dmac032 PMC9825273

[27] Zhou H Xu M Hu P. Identifying hub genes and common biological pathways between COVID-19 and benign prostatic hyperplasia by machine learning algorithms. Front Immunol. 2023;14:1172724. 37426635 10.3389/fimmu.2023.1172724 PMC10328422

[28] Hemani G Zheng J Elsworth B. The MR-Base platform supports systematic causal inference across the human phenome. Elife. 2018;7:e34408. 29846171 10.7554/eLife.34408 PMC5976434

[29] Xu HY Zhang YQ Liu ZM. ETCM: an encyclopaedia of traditional Chinese medicine. Nucleic Acids Res. 2019;47:D976–82. 30365030 10.1093/nar/gky987 PMC6323948

[30] Ru J Li P Wang J. TCMSP: a database of systems pharmacology for drug discovery from herbal medicines. J Cheminform. 2014;6:13. 24735618 10.1186/1758-2946-6-13 PMC4001360

[31] Fang S Dong L Liu L. HERB: a high-throughput experiment- and reference-guided database of traditional Chinese medicine. Nucleic Acids Res. 2021;49:D1197–206. 33264402 10.1093/nar/gkaa1063 PMC7779036

[32] Daina A Michielin O Zoete V. SwissTargetPrediction: updated data and new features for efficient prediction of protein targets of small molecules. Nucleic Acids Res. 2019;47:W357–64. 31106366 10.1093/nar/gkz382 PMC6602486

[33] Stelzer G Rosen N Plaschkes I. The genecards suite: from gene data mining to disease genome sequence analyses. Curr Protoc Bioinformatics. 2016;54:1.30.1–1.30.33. 10.1002/cpbi.5 27322403

[34] Piñero J Bravo A Queralt-Rosinach N. DisGeNET: a comprehensive platform integrating information on human disease-associated genes and variants. Nucleic Acids Res. 2017;45:D833–9. 27924018 10.1093/nar/gkw943 PMC5210640

[35] Gustavsson EK Zhang D Reynolds RH Garcia-Ruiz S Ryten M. ggtranscript: an R package for the visualization and interpretation of transcript isoforms using ggplot2. Bioinformatics. 2022;38:3844–6. 35751589 10.1093/bioinformatics/btac409 PMC9344834

[36] Gu Z Hübschmann D. Make interactive complex heatmaps in R. Bioinformatics. 2022;38:1460–2. 34864868 10.1093/bioinformatics/btab806 PMC8826183

[37] Mao W Ding J Li Y Huang R Wang B. Inhibition of cell survival and invasion by Tanshinone IIA via FTH1: a key therapeutic target and biomarker in head and neck squamous cell carcinoma. Exp Ther Med. 2022;24:521. 35837069 10.3892/etm.2022.11449 PMC9257971

[38] Shannon P Markiel A Ozier O. Cytoscape: a software environment for integrated models of biomolecular interaction networks. Genome Res. 2003;13:2498–504. 14597658 10.1101/gr.1239303 PMC403769

[39] Wu T Hu E Xu S. clusterProfiler 4.0: a universal enrichment tool for interpreting omics data. Innovation (Camb). 2021;2:100141. 34557778 10.1016/j.xinn.2021.100141 PMC8454663

[40] Szklarczyk D Kirsch R Koutrouli M. The STRING database in 2023: protein-protein association networks and functional enrichment analyses for any sequenced genome of interest. Nucleic Acids Res. 2023;51:D638–46. 36370105 10.1093/nar/gkac1000 PMC9825434

[41] Burgess S Thompson SG. Interpreting findings from Mendelian randomization using the MR-Egger method. Eur J Epidemiol. 2017;32:377–89. 28527048 10.1007/s10654-017-0255-x PMC5506233

[42] Bowden J Smith GD Haycock PC Burgess S. Consistent estimation in mendelian randomization with some invalid instruments using a weighted median estimator. Genet Epidemiol. 2016;40:304–14. 27061298 10.1002/gepi.21965 PMC4849733

[43] Burgess S Scott RA Timpson NJ Smith GD Thompson SG. Using published data in Mendelian randomization: a blueprint for efficient identification of causal risk factors. Eur J Epidemiol. 2015;30:543–52. 25773750 10.1007/s10654-015-0011-z PMC4516908

[44] Hu J Song J Chen Z. Reverse causal relationship between periodontitis and shortened telomere length: bidirectional two-sample Mendelian random analysis. Front Immunol. 2022;13:1057602. 36601105 10.3389/fimmu.2022.1057602 PMC9806346

[45] Berman HM Westbrook J Feng Z. The protein data bank. Nucleic Acids Res. 2000;28:235–42. 10592235 10.1093/nar/28.1.235 PMC102472

[46] Park S Lee KS Choi M Lee M. Factors associated with quality of life in patients with benign prostatic hyperplasia, 2009-2016. Medicine (Baltim). 2022;101:e30091. 10.1097/MD.0000000000030091 PMC9512327 36086750

[47] Batista-Miranda JE Diez MD Bertrán PA Villavicencio H. Quality-of-life assessment in patients with benign prostatic hyperplasia: effects of various interventions. PharmacoEcon. 2001;19:1079–90. 10.2165/00019053-200119110-00002 11735675

[48] Kim SK Park HK Choi HS Yoo KH Chung JH. Association study of polymorphisms of epidermal growth factor and epidermal growth factor receptor with benign prostatic hyperplasia in a Korean Population. Int Neurourol J. 2016;20:363–70. 28043105 10.5213/inj.1632538.269 PMC5209572

[49] Zeng F Harris RC. Epidermal growth factor, from gene organization to bedside. Semin Cell Dev Biol. 2014;28:2–11. 24513230 10.1016/j.semcdb.2014.01.011 PMC4037350

[50] Burgess AW. Regulation of signaling from the epidermal growth factor family. J Phys Chem B. 2022;126:7475–85. 36169380 10.1021/acs.jpcb.2c04156

[51] Hennenberg M Schreiber A Ciotkowska A. Cooperative effects of EGF, FGF, and TGF-β1 in prostate stromal cells are different from responses to single growth factors. Life Sci. 2015;123:18–24. 25529149 10.1016/j.lfs.2014.12.006

[52] De Bellis A Ghiandi P Comerci A. Epidermal growth factor, epidermal growth factor receptor, and transforming growth factor-alpha in human hyperplastic prostate tissue: expression and cellular localization. J Clin Endocrinol Metab. 1996;81:4148–54. 8923874 10.1210/jcem.81.11.8923874

[53] Sciarra F. Sex steroids and epidermal growth factor in benign prostatic hyperplasia (BPH). Ann N Y Acad Sci. 1995;761:66–78. 7542859 10.1111/j.1749-6632.1995.tb31370.x

[54] Nascimento-Viana JB Alcántara-Hernández R Oliveira-Barros E. The α1-adrenoceptor-mediated human hyperplastic prostate cells proliferation is impaired by EGF receptor inhibition. Life Sci. 2019;239:117048. 31730867 10.1016/j.lfs.2019.117048

[55] Gregory CW Fei X Ponguta LA. Epidermal growth factor increases coactivation of the androgen receptor in recurrent prostate cancer. J Biol Chem. 2004;279:7119–30. 14662770 10.1074/jbc.M307649200

[56] Izumi K Zheng Y Li Y Zaengle J Miyamoto H. Epidermal growth factor induces bladder cancer cell proliferation through activation of the androgen receptor. Int J Oncol. 2012;41:1587–92. 22922989 10.3892/ijo.2012.1593 PMC3583640

[57] Léotoing L Manin M Monté D. Crosstalk between androgen receptor and epidermal growth factor receptor-signalling pathways: a molecular switch for epithelial cell differentiation. J Mol Endocrinol. 2007;39:151–62. 17693613 10.1677/JME-07-0021

[58] Park JE Shin WC Lee HJ. SH-PRO extract alleviates benign prostatic hyperplasia via ROS-mediated activation of PARP/caspase 3 and inhibition of FOXO3a/AR/PSA signaling in vitro and in vivo. Phytother Res. 2023;37:452–63. 36122906 10.1002/ptr.7626

[59] Lin J Zhou J Xu W Zhong X Hong Z Peng J. Qianliening capsule treats benign prostatic hyperplasia via suppression of the EGF/STAT3 signaling pathway. Exp Ther Med. 2013;5:1293–300. 23737867 10.3892/etm.2013.1008 PMC3671788

[60] Wei H Wu G Shi D. Total flavan glycoside from Abacopteris penangiana rhizomes and its acid hydrolysate: characterisation and anti-benign prostatic hyperplasia potential. Food Chem. 2012;134:1959–66. 23442644 10.1016/j.foodchem.2012.03.128

[61] Elbaz EM Darwish A Gad AM Abdel Rahman AAS Safwat MH. Canagliflozin alleviates experimentally induced benign prostate hyperplasia in a rat model: exploring potential mechanisms involving mir-128b/EGFR/EGF and JAK2/STAT3 signaling pathways through in silico and in vivo investigations. Eur J Pharmacol. 2023;957:175993. 37598927 10.1016/j.ejphar.2023.175993

[62] Zhang Q Tian Y Fu Z. The role of serum-glucocorticoid regulated kinase 1 in reproductive viability: implications from prenatal programming and senescence. Mol Biol Rep. 2024;51:376. 38427115 10.1007/s11033-024-09341-8 PMC10907440

[63] Maestro I Boya P Martinez A. Serum- and glucocorticoid-induced kinase 1, a new therapeutic target for autophagy modulation in chronic diseases. Expert Opin Ther Targets. 2020;24:231–43. 32067528 10.1080/14728222.2020.1730328

[64] Cicenas J Meskinyte-Kausiliene E Jukna V Rimkus A Simkus J Soderholm D. SGK1 in cancer: biomarker and drug target. Cancers (Basel). 2022;14:2385. 35625991 10.3390/cancers14102385 PMC9139822

[65] Howard PG Zou P Zhang Y. Serum/glucocorticoid regulated kinase 1 (SGK1) in neurological disorders: pain or gain. Exp Neurol. 2024;382:114973. 39326820 10.1016/j.expneurol.2024.114973 PMC11536509

[66] Lin D Luo C Wei P. YAP1 recognizes inflammatory and mechanical cues to exacerbate benign prostatic hyperplasia via promoting cell survival and fibrosis. Adv Sci (Weinh). 2024;11:e2304274. 38050650 10.1002/advs.202304274 PMC10837380

[67] Li Y Zhou Y Liu D. Glutathione Peroxidase 3 induced mitochondria-mediated apoptosis via AMPK/ERK1/2 pathway and resisted autophagy-related ferroptosis via AMPK/mTOR pathway in hyperplastic prostate. J Transl Med. 2023;21:575. 37633909 10.1186/s12967-023-04432-9 PMC10463608

[68] Yang T Shao Y Wang Z Liu C Gu M. Epigallocatechin-3-gallate attenuates benign prostatic hyperplasia development via regulating firmicutes to inhibit gastric secretion of insulin-like growth factor-1. Cell Biol Int. 2025;49:952–64. 40552775 10.1002/cbin.70032

[69] Jang H Park Y Jang J. Serum and glucocorticoid-regulated kinase 1: Structure, biological functions, and its inhibitors. Front Pharmacol. 2022;13:1036844. 36457711 10.3389/fphar.2022.1036844 PMC9706101

[70] Xiao Q Liu QM Jiang RC. Piperazine-Derived α1D/1A Antagonist 1- Benzyl-N- (3-(4- (2-Methoxyphenyl) Piperazine-1-yl) Propyl) -1H- Indole-2- carboxamide induces apoptosis in benign prostatic hyperplasia independently of α1-adrenoceptor blocking. Front Pharmacol. 2021;11:594038. 33584271 10.3389/fphar.2020.594038 PMC7873900

[71] Inamura S Terada N. Chronic inflammation in benign prostatic hyperplasia: pathophysiology and treatment options. Int J Urol. 2024;31:968–74. 38934050 10.1111/iju.15518 PMC11524144

[72] Kai W Lin C Jin Y. Urethral meatus stricture BOO stimulates bladder smooth muscle cell proliferation and pyroptosis via IL 1β and the SGK1 NFAT2 signaling pathway. Mol Med Rep. 2020;22:219–26. 32468047 10.3892/mmr.2020.11092 PMC7248470

[73] Roberts F Zhu D Farquharson C Macrae VE. ENPP1 in the regulation of mineralization and beyond. Trends Biochem Sci. 2019;44:616–28. 30799235 10.1016/j.tibs.2019.01.010

[74] Onyedibe KI Wang M Sintim HO. ENPP1, an old enzyme with new functions, and small molecule inhibitors-A STING in the Tale of ENPP1. Molecules. 2019;24:4192. 31752288 10.3390/molecules24224192 PMC6891441

[75] Chauvin SD Stinson WA Platt DJ Poddar S Miner JJ. Regulation of cGAS and STING signaling during inflammation and infection. J Biol Chem. 2023;299:104866. 37247757 10.1016/j.jbc.2023.104866 PMC10316007

[76] Xu J Chen C Yin J. Lactate-induced mtDNA Accumulation Activates cGAS-STING signaling and the inflammatory response in sjögren’s syndrome. Int J Med Sci. 2023;20:1256–71. 37786436 10.7150/ijms.83801 PMC10542019

[77] Naiyila X Li J Huang Y. A novel insight into the immune-related interaction of inflammatory cytokines in benign prostatic hyperplasia. J Clin Med. 2023;12:1821. 36902608 10.3390/jcm12051821 PMC10003138

[78] Zhou M Gong X Ru Q. The neuroprotective effect of l-stepholidine on methamphetamine-induced memory deficits in mice. Neurotox Res. 2019;36:376–86. 31201732 10.1007/s12640-019-00069-z

[79] Hicks C Huang P Ramos L. Dopamine D1-Like receptor agonist and D2-Like receptor antagonist (-)-Stepholidine reduces reinstatement of drug-seeking behavior for 3,4-Methylenedioxypyrovalerone (MDPV) in Rats. ACS Chem Neurosci. 2018;9:1327–37. 29597343 10.1021/acschemneuro.7b00510 PMC6062002

[80] Cho KJ Koh JS Choi J Kim JC. Changes in adenosine triphosphate and nitric oxide in the urothelium of patients with benign prostatic hyperplasia and detrusor underactivity. J Urol. 2017;198:1392–6. 28655527 10.1016/j.juro.2017.06.080

[81] Silva-Ramos M Silva I Faria M. Impairment of ATP hydrolysis decreases adenosine A1 receptor tonus favoring cholinergic nerve hyperactivity in the obstructed human urinary bladder. Purinergic Signal. 2015;11:595–606. 26521170 10.1007/s11302-015-9478-z PMC4648796

