# Supplementary figures and images for: Identification of biomarkers of Shenhailong formula in benign prostatic hyperplasia treatment: An observation study using network pharmacology and Mendelian randomization analysis
Source: Medicine (Baltimore). 2025 Nov 21;104(47):e45619. doi: 10.1097/MD.0000000000045619 (PMC12643769; doi:10.1097/MD.0000000000045619)

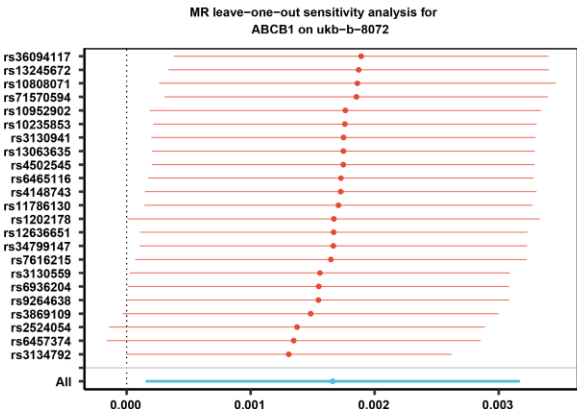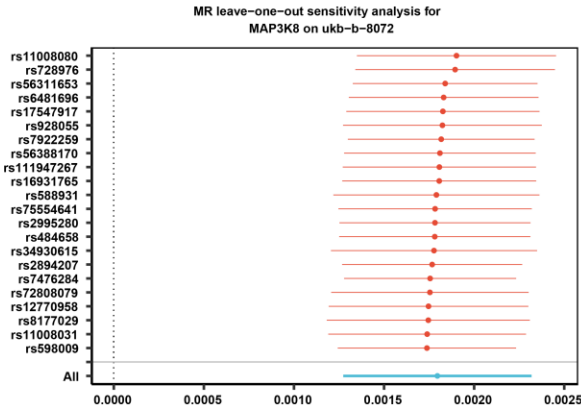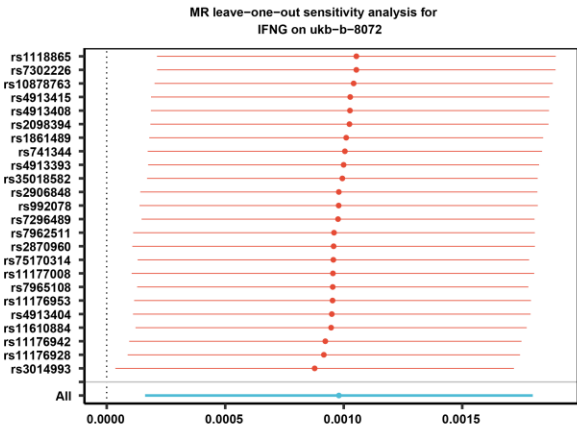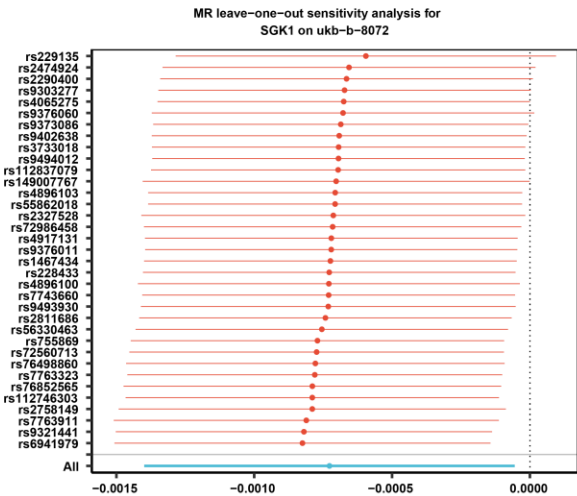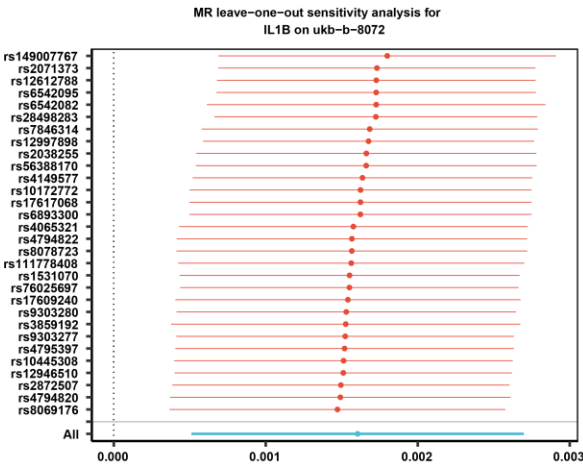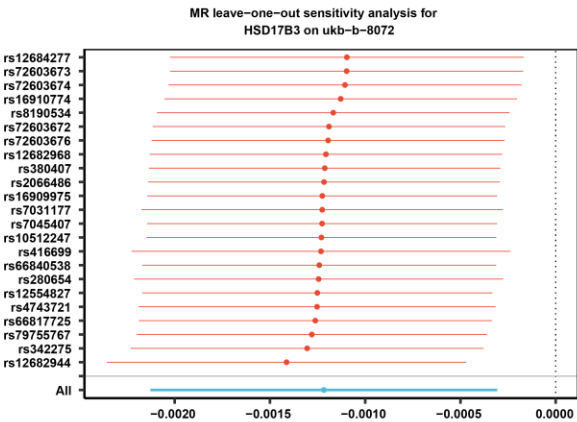



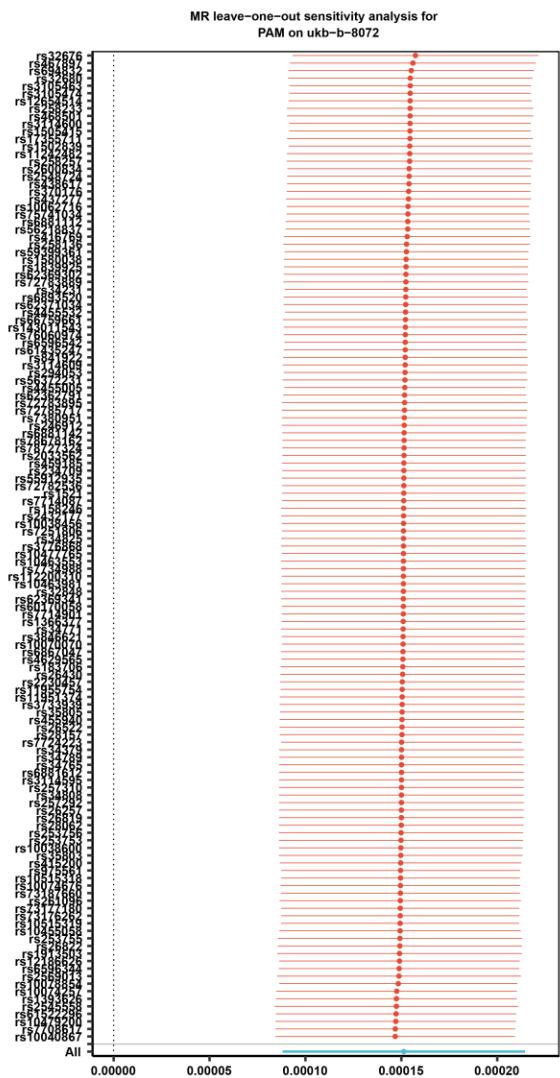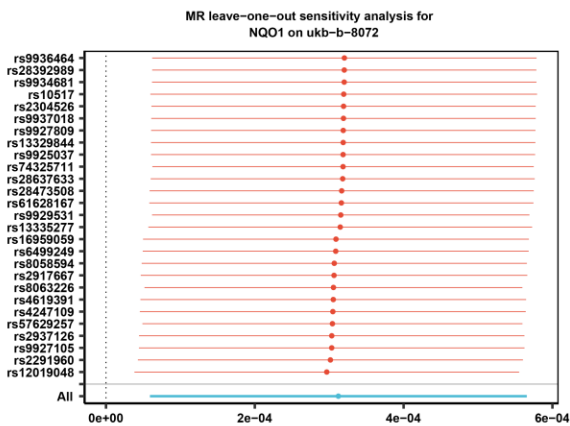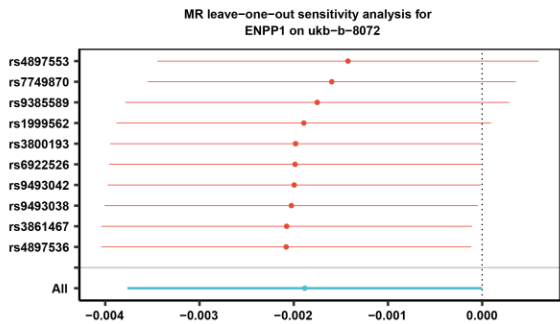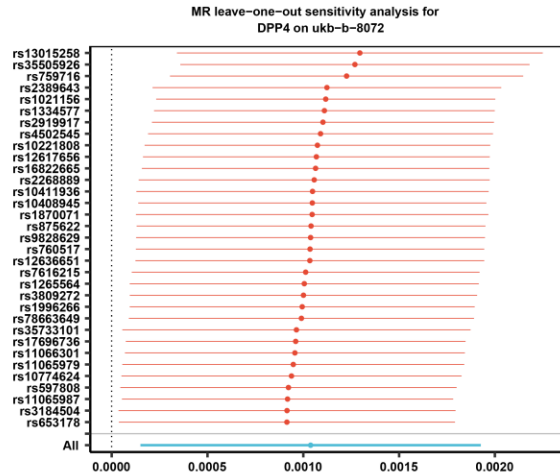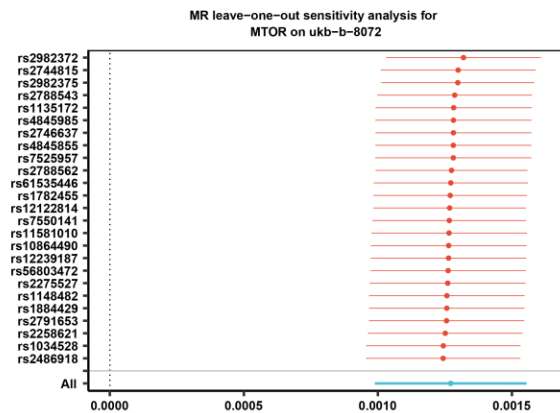

TrainDat Expression

group control Case

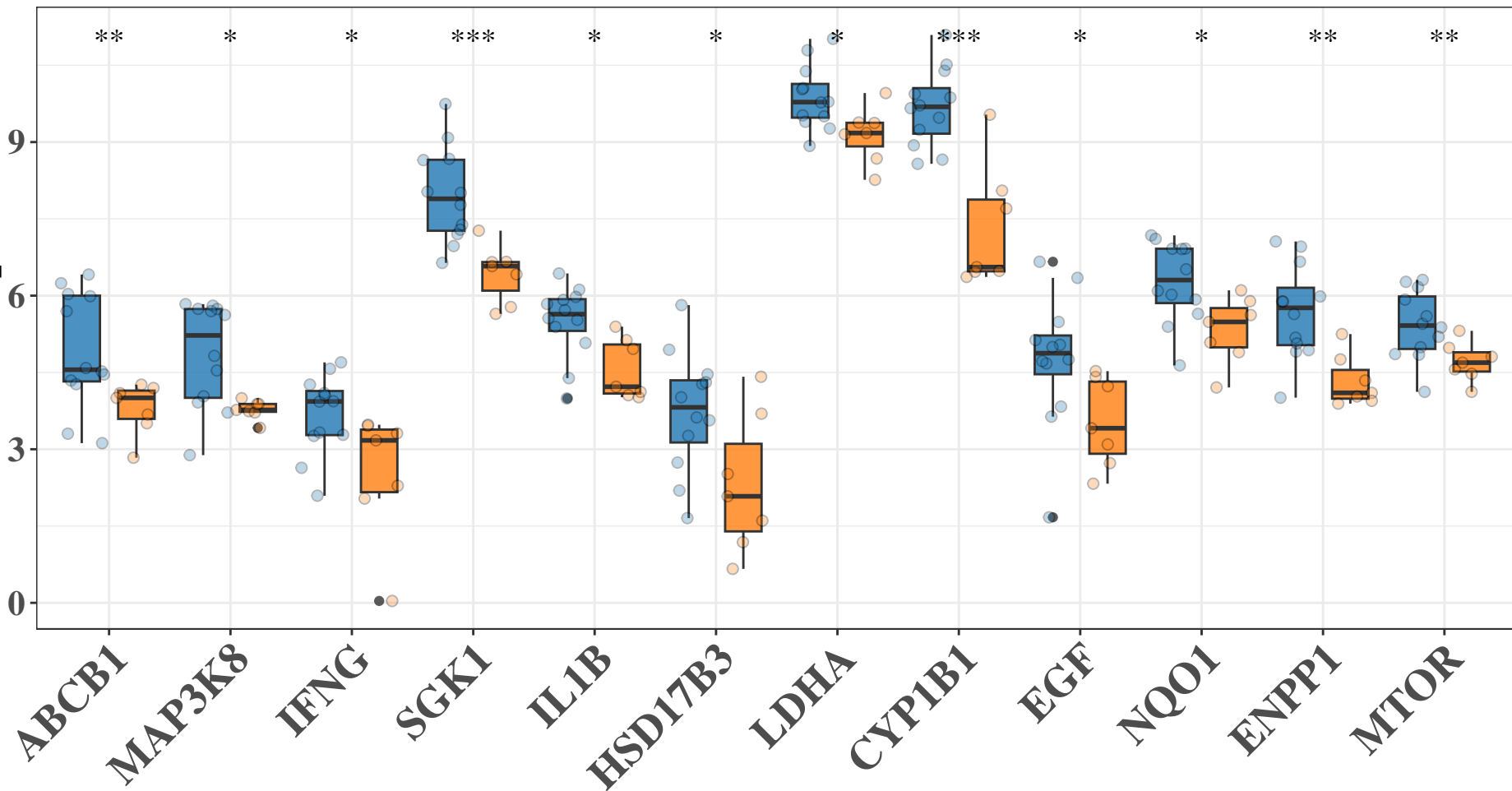

group control Case

VerifyDat Expression

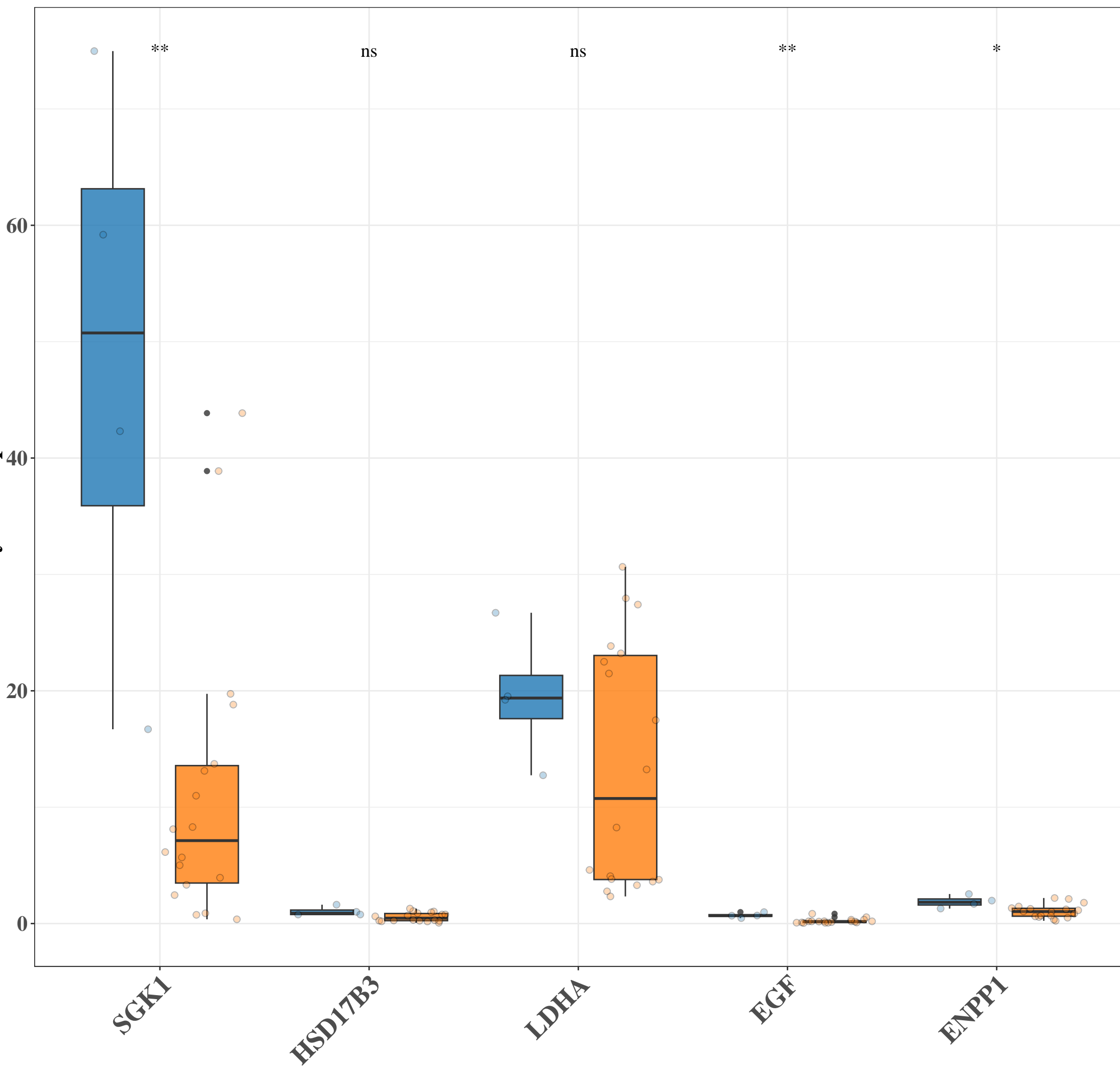

Supplement: Supplementary file 2 [file medi-104-e45619-s002.pdf]
